# Acute Ischemic Stroke in COVID-19: Putative Mechanisms, Clinical Characteristics, and Management

**DOI:** 10.1155/2020/7397480

**Published:** 2020-11-04

**Authors:** Ademola S. Ojo, Simon A. Balogun, Ahmed O. Idowu

**Affiliations:** ^1^Department of Anatomical Sciences, St. George's University School of Medicine, St. George's, Grenada; ^2^Department of Neurological Surgery, Obafemi Awolowo University Teaching Hospital Complex, Ile Ife, Nigeria; ^3^Neurology Division, Department of Medicine, Obafemi Awolowo University Teaching Hospital Complex, Ile Ife, Nigeria

## Abstract

The emergence and spread of the highly contagious novel coronavirus disease (COVID-19) have triggered the greatest public health challenge of the last century. Aside from being a primary respiratory disease, acute ischemic stroke has emerged as a complication of the disease. While current evidence shows COVID-19 could cause ischemic stroke especially in severe disease, there are similarities in the risk factors for severe COVID-19 as well as ischemic stroke, underscoring the complex relationship between these two conditions. The pandemic has created challenges for acute stroke care. Rapid assessment and time-sensitive interventions required for optimum outcomes in acute stroke care have been complicated by COVID-19 due to the need for disease transmission preventive measures. The purpose of this article is to explore the putative mechanisms of ischemic stroke in COVID-19 and the clinical characteristics of COVID-19 patients who develop ischemic stroke. In addition, we discuss the challenges of managing acute ischemic stroke in the setting of COVID-19 and review current management guidelines. We also highlighted potential areas for future research.

## 1. Introduction

The emergence and spread of the highly contagious novel coronavirus disease (COVID-19) in late 2019 have triggered the greatest public health challenge of the last century. With over 6,000,000 confirmed cases and more than 300,000 deaths in the first five months, COVID-19 has established itself as the worst infectious disease outbreaks in recent history. Although it is considered a primary respiratory disease, there is evidence of multisystemic involvement including neurological manifestations [1]. Hypogeusia, anosmia, seizure, and stroke are some of the reported neurological features of the disease [2]. While the exact relationship between stroke and COVID-19 is still emerging, there is growing evidence that the disease process may trigger an ischemic stroke or worsen an existing stroke [3]. Risk factors such as male sex, advanced age, and the presence of comorbidities such as hypertension, diabetes, and heart diseases are predictors of severe COVID-19 [4]. These factors are also predisposing factors for stroke, which underlies the complex relationship between these two conditions. In addition, the current pandemic has direct and indirect implications for stroke care. Stroke patients are at increased odds of severe disease when infected with COVID-19 [5]. Also, patients with COVID-19 who develop stroke are at a higher risk of a negative outcome when compared to non-COVID-19 stroke patients [6]. On the other hand, the strain on available healthcare resources caused by the pandemic could negatively impact stroke care delivery services, thereby limiting stroke patients' access to prompt and time-sensitive intervention [3]. This review, therefore, explores the putative mechanisms of ischemic stroke in COVID-19 and the clinical characteristics of stroke in COVID-19 patients. In addition, we review the current guidelines on the management and challenges of managing patients with acute ischemic stroke in the setting of COVID-19.

## 2. Pathogenesis of Ischemic Stroke in COVID-19

Severe acute respiratory syndrome coronavirus 2 (SARS-CoV-2) is an enveloped, single-stranded, positive-sense RNA virus of the Coronaviridae family and it has a phospholipid bilayer capsule containing spike proteins [7]. SARS-CoV-2 gains access to the body through the interaction of its spike (S) protein and angiotensin-converting enzyme 2 (ACE2), found in many tissues such as lung, kidney, heart, and intestines [7]. While details of the pathogenesis of ischemic stroke in COVID-19 are still emerging, current evidence suggests putative mechanisms.

### 2.1. Coagulopathy

Current evidence shows that coagulopathy with increased risk of thromboembolic complications occurs in COVID-19 patients. “Sepsis-induced coagulopathy” (SIC) has emerged as a feature of severe COVID-19 disease [8]. SIC is an earlier phase of disseminated intravascular coagulopathy (DIC) characterized by elevated D-dimer, prolonged prothrombin time, and low platelet count, but, unlike DIC, hypofibrinogenemia is absent [9]. This follows systemic inflammatory response syndrome triggered by an immune response to the virus leading to endothelial dysfunction and thrombosis of the microcirculation [10]. In addition, there is oversuppression of fibrinolysis from increased production of plasminogen activator inhibitor-1, thereby increasing the risk of thrombosis [11]. Unlike DIC, there is no bleeding. Arterial and venous thrombosis is increasingly reported in COVID-19 patients [12].

In addition, the presence of antiphospholipid (APL) antibodies has long been linked with a prothrombotic state. In a study of 56 patients with confirmed COVID-19, 14 patients tested positive for lupus anticoagulant, while five had anti-cardiolipin or anti-*β*_2_‐glycoprotein-1 *I*gM or *I*gG antibodies [13]. APL antibodies are associated with arterial and venous thrombosis [10]. However, APL antibodies are often found in healthy individuals, and elevated levels have been reported in both viral and bacterial infections [14]. It is still unclear if the presence of APL antibodies in COVID-19 patients contributes to the coagulopathy or increases the risk of ischemic stroke.

### 2.2. Endothelial Dysfunction

Endothelial dysfunction predisposes to the formation of arterial and venous thrombosis. Normal vascular endothelium has pro- and antithrombotic factors that are normally in a state of equilibrium to prevent bleeding or clot formation. One possible mechanism of endothelial dysfunction in patients with COVID-19 is the depletion of ACE2 receptors, which is found in vascular endothelial cells [9]. The homolog of ACE2 and ACE1 is an integral part of the renin-angiotensin-aldosterone system [9]. This system has a classical arm and renin/ACE1/angiotensin II/angiotensin II type 1 receptor axis which has physiologic and pathologic roles in diseases of the kidney, blood vessels, heart, and brain and the ACE2/angiotensin (1–7)/mitochondrial assembly receptor (MasR) axis, which has been identified as a negative regulator of angiotensin II [15]. Renin is produced by the renal juxtaglomerular cells, and it converts angiotensinogen produced by the liver to angiotensin I. ACE1 cleaves angiotensin I to angiotensin II. Angiotensin II acting through its receptors has vasopressor, proinflammatory, and procoagulant effects [9]. Inhibitors of angiotensin II receptors have been shown to have antihypertensive as well as neuroprotective effects [16]. ACE2 counteracts the action of ACE1 by converting angiotensin II to angiotensin (1–7), a vasodilator and anti-inflammatory molecule, and converting angiotensin I to angiotensin (1–9), which is further converted to angiotensin (1–7) [17]. Angiotensin (1–7) mediates its effect through the Mas receptor. By binding to ACE2, SARS-CoV-2 induces receptor endocytosis, thereby leading to depletion of the “protective” endothelial ACE2 and tilting the balance in favor of ACE1 and angiotensin II, with an increased proinflammatory disposition and endothelial injury [9].

Another possible mechanism of endothelial injury in patients with COVID-19 is the immune hyper reaction referred to as a cytokine storm [18]. This uncontrolled and excessive release of cytokines in COVID-19 has been described as a major contributor to multiple organ dysfunction [18]. Elevated levels of cytokines such as IL1-*β*, IL-7, IL-8, IL-9, IL-10, granulocyte-macrophage colony-stimulating factor (GMCSF), IFN-*γ*, monocyte chemoattractant protein (MCP1), and tumor necrosis factor-*α* (TNF*α*) has been reported in COVID-19 [19]. Inflammatory cytokines cause endothelial activation, increased expression of endothelial leukocyte adhesion molecules (ELAMs) such as intercellular adhesion molecule 1 (ICAM-1), E-selectin, and vascular cell adhesion molecule 1 (VCAM-1) which interact with leukocyte surface receptors [20]. The effect of the cytokines and leukocyte-mediated injury could disrupt the integrity of the endothelial cells. Details of how much this process contributes to ischemic stroke in COVID-19 patients are still emerging.

### 2.3. Cardioembolism

Cardiac injury has been reported as one of the leading causes of death in COVID-19 after respiratory dysfunction [21]. Elevated markers of cardiac injury such as troponin I&T and creatinine kinase-MB reported in COVID-19 patients have been shown to correlate with disease severity [21]. Direct virus-mediated injury and the effect of the cytokine storm has been proposed as being responsible for cardiac injury in COVID-19 [22]. This manifests with acute myocarditis, pericarditis, arrhythmia, shock, and cardiac failure [22]. Cardiac endothelial dysfunction and arrhythmia increase the risk of cardiac thromboembolism and stroke. The role of this in ischemic stroke in COVID-19 is still hypothetical at this point.

### 2.4. Direct Viral Invasion of the Central Nervous System

Two potential channels of CNS invasion have been proposed for SARS-CoV-2: (1) hematogenous spread to the cerebral circulation from systemic dissemination and (2) transmission through the olfactory epithelium via the cribriform plate to the olfactory bulb [2]. As the virus is present in the general circulation including the cerebral circulation, the sluggish cerebral microcirculation enhances the interaction between the virus spike protein and ACE2 on cerebral endothelial cells [23]. Viral proliferation and release from endothelial cells could lead to endothelial damage and allow viral access to the brain [23]. Also, ACE2 receptors have been detected on neurons and glial cells, making them a potential target for SARS-CoV-2 [24]. While the exact mechanism of transcribiform invasion of SARS-CoV-2 is not fully understood, a rodent model of SARS-CoV-1 infection, a close “cousin” of SARS-CoV-2, shows the presence of the virus in areas of the brain connected to the olfactory bulb after transnasal exposure which is associated with neuronal death [24]. The presence of anosmia, hypogeusia, and other neurological findings in COVID-19 patients support the theory of a direct effect of the virus on the brain [23]. The exact role this plays in ischemic stroke is however still emerging.

### 2.5. Immunoglobulin Therapy

The lack of an effective vaccine or therapy for COVID-19 has led to the trial of convalescent plasma in the treatment of critically ill patients with COVID-19 [25]. Intravenous immunoglobulin (IVIg) therapy has been used in both postexposure prophylaxis and treatment of virus infections [25]. However, IVIg therapy has potential complications. The risk of an intravenous immunoglobulin-associated thromboembolic event is estimated at 0.6 to 4.5% [26]. Events such as stroke, myocardial infarction, pulmonary embolism, and deep venous thrombosis are thought to be due to IVIg-induced increase in blood viscosity, thereby predisposing to stasis, a risk factor for thrombus formation [26]. In a study of 206 patients with severe acute respiratory distress syndrome (SARS), five patients developed large-artery cerebral infarctions with three of them occurring in patients who received IVIg therapy [27]. Critically ill patients are at increased risk of thrombosis, as such, the use of IVIg in these patients could further worsen the risk of thromboembolic events. Currently, there is limited evidence to support the role of IVIg therapy in ischemic stroke in patients with COVID-19.

## 3. Clinical Characteristics of Patients with COVID-19 and Stroke

There have been reports of a stroke occurring in COVID-19 patients. In a retrospective study of 3556 COVID-19 patients in a New York hospital system, 32 (0.9%) developed ischemic stroke [6]. 65.6% (21/32) had a cryptogenic stroke and 34.4% (11/32) met the criteria for an embolic stroke of undetermined source. When compared with stroke patients without COVID-19, patients with COVID-19 were older (median age-70 versus 63 years, *P*=0.001), had higher national institute of health stroke score (NIHSS) at admission (median score-19 versus 8, *P*=0.007), with elevated peak D-dimer level (>10 000 versus 525 ng/mL, *P*=0.011), and were more likely to have a cryptogenic stroke (65.6% versus 34.4%, *P*=0.003) and at a higher risk of inpatient mortality (63.6% versus 9.3%, *P*=0.001). 25 of the patients developed stroke within 1–27 days of disease onset, while five were previously asymptomatic.

Similarly, a single-center retrospective study involving 221 COVID-19 patients showed that 11 (5%) developed acute ischemic stroke, with cerebral venous thrombosis occurring in one patient while another developed cerebral hemorrhage [28]. When compared to other COVID-19 patients without stroke, those who developed stroke were older (mean age-72 versus 52 years) and had a higher frequency of comorbidities such as hypertension, diabetes, previous stroke history, and hepatic and renal dysfunction. Large-artery disease, medium-vessel disease, and cardioembolic events were reported in five, three, and three patients, respectively. D-dimer was 12 times higher in those who developed stroke. The patients developed stroke within 1–29 days of infection (mean-10 days).

Although stroke occurs more commonly in older individuals, younger patients with stroke have been reported in COVID-19. In the previously mentioned study, stroke was more likely to occur in younger men with elevated serum troponin levels when compared to historical controls [6]. Similarly, Oxley and colleagues reported five cases of large-vessel stroke in COVID-19 patients younger than 50 years [29]. The vascular territories involved in imaging in the cases are the middle cerebral artery territory (three), posterior cerebral artery (one), and internal carotid artery (one) [29]. Three of the five patients have COVID-19 symptoms, while two are asymptomatic. Similarly, a study reported three COVID-19 patients with large-vessel ischemic stroke following subocclusive severe stenosis of the common carotid artery with the thrombosis extending into the proximal internal carotid artery [30]. The three patients were 33, 55, and 77 years. Another study reported a case of a 40-year-old COVID-19 patient with a negative procoagulant work-up who developed large-vessel ischemic stroke involving the middle cerebral artery [31]. A similar report of large-artery ischemic stroke was found in five out of 206 patients with SARS in a study [27]. In that study, four of the patients were above 50 years (54–68), while the 5th patient was 39 years. The link between large-artery ischemic stroke and COVID-19 in younger patients is not fully understood; however, it may be related to hypercoagulability in these patients.

In a retrospective study of 214 COVID-19 patients in Wuhan, China, 5.7% developed stroke. These findings were reported in patients with severe disease [32]. Markers of severe disease in COVID-19 include lymphopenia, leukocytosis, hypoalbuminemia as well as elevated levels of alanine transaminase, C-reactive protein, ferritin, lactate dehydrogenase, and D-dimer [33]. In a study reporting six patients with COVID-19 who developed ischemic stroke, all the patients have large-vessel occlusion with markedly elevated D-dimers. Lupus anticoagulants, as well as C-reactive protein and ferritin, were elevated in five of the patients [34]. Similarly, in a study of four patients with ischemic stroke, three had elevated D-dimer and two have elevated C-reactive protein levels [35].

The outcome of stroke in COVID-19 patients may reflect disease severity. The National Institute of Health Stroke Score (NIHSS) has been used to predict stroke outcome. Those with higher scores are at risk of poor outcomes. When compared with stroke patients who did not have COVID-19, patients with stroke and COVID-19 were reported to have a higher NIHSS score [6]. In the study by Oxley and colleagues, all five patients with large-vessel stroke had a score of 13–23, predicting a higher risk of poor disease outcome [29]. Similarly, in a retrospective study of six COVID-19 patients with stroke, five died, while one had persistent severe neurological deficit [36].

## 4. Challenges of Managing Stroke in the Setting of COVID-19

COVID-19, directly and indirectly, affect stroke care. Since the outbreak of the disease, many measures have been imposed to combat the scourge of this infection such as lockdown measures, suspending elective procedures, and routine outpatient clinics, as well as redeployment of healthcare resources [37]. According to the World Stroke Organization (WSO), many countries have experienced overwhelmed hospital capacity which results in the reallocation of neurology and stroke beds as well as intensive care unit (ICU) facilities meant for stroke care to caring for COVID-19 patients [3]. In addition, the redeployment of stroke physicians and nurses to COVID-19 care occurred in many hospitals [38]. The effect of this is a reduced ability to provide the necessary care for stroke patients. An observatory study involving 280 centers in China shows hospital admissions for stroke reduced by 40%, while thrombolysis and thrombectomy cases dropped by 25% in February 2020, when compared to February 2019 [39]. This reduction in stroke admissions could be due to patients opting to stay at home out of fear of contracting the virus in the hospital. The overall effect of this is a delayed onset of care, with many patients missing the therapeutic window [3].

In addition, the risk of exposure to COVID-19 increases in healthcare providers who have contact with infected patients. Globally, there have been reports of COVID-19 infection and death among health care workers [38]. This necessitates the use of the personal protective equipment, which often is in short supply in some countries. Infection among stroke care team members who are also in short supply due to redeployment could leads to quarantine or even hospital admission, further depleting the human resources available for stroke care.

## 5. Management of Acute Ischemic Stroke in COVID-19

Prior to the current global pandemic, stroke care guidelines have been developed and universally adopted by healthcare professionals involved in acute stroke management [40]. One such is the guideline for the management of acute ischemic stroke developed by the American Heart Association/American Stroke Association (AHA/ASA) [40]. This document provides guidance on prehospital stroke management based on robust stroke systems, emergency evaluation, and treatment, as well as general supportive care (readers are referred to the guideline for more information). Due to the time-dependent nature of acute ischemic stroke, current stroke guidelines recommend emergency care should be administered rapidly while avoiding any unnecessary time-wasting intervention [41]. All suspected stroke cases should have prompt brain imaging on hospital arrival, preferably within 20 mins of hospital arrival [40]. Stroke patients who meet the criteria should undergo rapid interventions with thrombolysis using recombinant tissue plasminogen activator (alteplase) and/or mechanical thrombectomy in those with large-vessel occlusion. Interventions should be carried out within the shortest possible time to limit brain damage [41]. This process of acute stroke care has been complicated since the emergence of COVID-19. In response to the challenges of managing acute stroke created by the pandemic, various consensus studies have come up with recommendations for the management of stroke in COVID-19 [3, 42, 43]. The American Heart Association/American Stroke Association also issued a temporary guideline for the management of stroke in patients with COVID-19 [44]. The common theme of these guidelines is summarized below.

### 5.1. Initial Triage and Evaluation

Whether a new patient presenting to the emergency department (ED) or one already on hospital admission, all stroke patients undergo an initial rapid assessment. This routine process is now complicated by COVID-19, necessitating factoring the infection status of the patient into consideration. Three categories of COVID-19 infected stroke patients have been described, which should be determined rapidly during the initial assessment: (1) patient with known infection who are usually in the advanced phase of the disease, (2) patients with suspected COVID-19 infection based on a history of disease symptoms, contact with infected individuals, and residence or travel to high-risk areas, and (3) those who are asymptomatic with no history of contact with infected individuals or residence in high-risk areas [42]. Patients who have a suspected or confirmed infection should be separated early. Where possible, this categorization should be carried out by the emergency medical services before arrival in the ED [41]. However, many stroke patients may be confused, aphasic, or unconscious, complicating the effort to determine the risk of infection. Also, asymptomatic individuals could be disease spreaders. It is, therefore, safe to entertain a high index of suspicion in all patients. This will ensure necessary protective measures are taken by healthcare personnel. Considering the urgent nature of acute stroke care and the time frame required to obtain a confirmatory test result for COVID-19 patients, disease transmission preventive measures such as the use of personal protective equipment (PPE), hand washing, and social distancing measures should be employed during this initial assessment [42]. If possible, remote assessment using telemedicine (telestroke) should be considered [40, 45]. This allows the remote evaluation of radiological images as well as video-assisted neurological examination, thereby limiting the exposure of both healthcare professionals and patients [41]. Compared to the bedside assessment, telestroke has been shown to be effective in assessing the NIHSS score, as well as eligibility for thrombolysis and thrombectomy [45].

### 5.2. Consider Organ Dysfunction

The outcome of stroke care in COVID-19 may mirror the severity of the underlying disease. The mortality rate in patients with stroke and COVID-19 has been shown to be higher than those without COVID-19 [29, 36]. It is therefore important to assess the predictors of increased in-hospital mortality. These factors include older age, cardiovascular diseases, acute kidney injury, high Sequential Organ Failure Assessment (SOFA) score, hypoalbuminemia, and elevated levels of transaminase, lactate dehydrogenase, ferritin, D-dimer, and C-reactive protein [33]. The SOFA score has been used as an index of severity in the critically ill as well as predicting the risk of mortality. This assessment of organ dysfunction is important in determining the disease prognosis while considering the appropriate acute stroke care plan [42].

### 5.3. Neurovascular Imaging

The AHA/ASA guideline recommends emergency brain imaging preferably within 20 mins of arrival at the ED. For patients with large-vessel occlusion who meet the criteria for thrombectomy, a computed tomography angiography (CTA) or CT perfusion studies are required depending on the disease onset-to-presentation time [40]. This guideline recommends proceeding with contrast studies before obtaining the serum creatinine level in a patient with no history of renal impairment [40]. In patients with COVID-19 and stroke, the risk of acute kidney injury is high. Contrast nephropathy could precipitate acute kidney injury, thereby worsening the mortality. There should be caution in the use of contrast studies in COVID-19 patients with stroke and overall-risk to benefit ratio should be considered [42]. In addition, a chest CT scan should be considered along with neurovascular imaging as this will give an insight into the extent of lung involvement [41]. Also, where possible, there should be a separate radiology suite dedicated to patients with a confirmed or high risk of COVID-19 with adequate decontamination procedure put in place [41].

### 5.4. Thrombolysis and Mechanical Thrombectomy

Current guidelines recommend commencing intravenous thrombolysis with rt-PA within 3 hours of stroke onset and within 3 to 4.5 hours in selected patients [40]. Hepatic dysfunction manifesting with elevated transaminases as well as coagulopathy is documented abnormalities in COVID-19 [33]. There is a higher risk of a negative outcome in stroke patients with elevated D-dimer levels who undergo intravenous thrombolysis [46]. Hepatic dysfunction in COVID-19 could occur with or without coagulopathy [42]. Assessment of hemostatic characteristics using tests such as thromboelastography and serum D-dimer level should be considered in these patients [42]. Although the impact of liver dysfunction on rt-PA metabolism in COVID-19 patients is not clear at this point, there is a potential risk of drug toxicity if hepatic metabolism is compromised. Stroke management teams should bear this in mind.

The current guideline recognizes the benefit of mechanical thrombectomy done within 6 hours of stroke onset in patients with large-vessel occlusion, NIHSS score ≥6, and who do not have extensive ischemia on CT scan [40]. In addition, selected stroke patients within 6–16 hours of last known normal and those within 6–24 hours who had CT perfusion studies or MRI are also considered reasonable choices for thrombectomy [40]. The specialized care required for thrombectomy might require an interhospital transfer, a process that could be complicated by additional logistic challenges due to COVID-19 [42]. Standard precaution protocols such as the use of PPE should be observed in angiography suites during mechanical thrombectomy to reduce the risk of exposure among healthcare personnel [41]. In addition, decontamination procedures for surfaces and equipment should be implemented [42]. If possible, the procedure should be carried out in a negative-pressure angiography suite fitted with a high-efficiency particulate air (HEPA) filter [46].

### 5.5. Supportive Care

Up to 38% of patients with acute ischemic stroke undergo intubation for reasons such as labored breathing, reduced level of consciousness, seizures, and high NIHSS score [47]. This may be higher in the setting of COVID-19. This procedure can create aerosols and, as such, it should be performed in an optimal setting with appropriate safety measures [42].

## 6. Conclusion and Recommendations for Future Research

Acute ischemic strokes are reported complications of COVID-19 with coagulopathy, endothelial dysfunction, cardioembolism, and direct viral-mediated neuronal injury as possible underlying causes. Older patients with severe disease are at a higher risk of this complication, but large-vessel occlusion is being commonly reported in younger patients. Rapid assessment and intervention required for optimum outcomes in hyperacute stroke care have been complicated by COVID-19. Infection control measures should be instituted while ensuring adequate care for these patients. There is, therefore, a need for more studies to further understand how COVID-19 causes stroke and the relationship between large-vessel stroke and younger age patients. In addition, the benefit of the use of anticoagulants in stroke prevention and treatment in COVID-19 patients should be explored.
